# New antibody-drug conjugates (ADCs) in breast cancer—an overview of ADCs recently approved and in later stages of development

**DOI:** 10.37349/etat.2022.00069

**Published:** 2022-02-24

**Authors:** Kira-Lee Koster, Jens Huober, Markus Joerger

**Affiliations:** 1 Medical Oncology and Hematology, Cantonal Hospital, CH-9000 St. Gallen, Switzerland; 2 Breast Center, Cantonal Hospital, CH-9000 St. Gallen, Switzerland; Institute of Oncology Research, Switzerland

**Keywords:** Antibody-drug conjugates, triple-negative breast cancer, human epidermal growth factor receptor 2-positive breast cancer

## Abstract

Antibody-drug conjugates (ADCs) have changed the treatment of breast cancer (BC) in more recent years. BC is a heterogenous group of malignancies with a broad range of histopathological characteristics. ADCs represent a class of therapeutics that combines an antigen-specific antibody backbone bound to a potent cytotoxic agent (the payload), via a linker, contributing to an improved therapeutic index. Currently, three ADCs received approval by the US Food and Drug Administration (FDA) and are in routine clinical use in different treatment settings; many more ADCs are in earlier and later stages of development, and their future approval will improve treatment options for patients with advanced but potentially also early-stage BC over time. Just recently, the results of three phase 3 trials (ASCENT, TULIP, and DESTINY-Breast03) evaluating sacituzumab govitecan (SG), trastuzumab duocarmazine, and trastuzumab deruxtecan (T-DXd) in different treatment settings were presented and showed promising results. This overview focuses on the newer ADCs, including T-DXd and SG, their pharmacology, mechanisms of action, and relevant studies. In addition, the latest results from trials investigating some newer ADCs, in further stages of development are presented.

## Introduction

Antibody-drug conjugates (ADCs) have changed the treatment of breast cancer (BC) in more recent years [1]. ADCs are a combination of an antibody targeting a specific antigen and a toxic payload conjugated with the antibody via a linker. This pharmacological modification of conventional monoclonal antibodies (mAbs) allows to bring a maximum of highly potent chemotoxic drugs into the cancer cell and reduces systemic toxicity to a certain degree. The antigen targeted by ADCs should be (over)expressed on the surface of the targeted cancer cells and should exhibit no or minimal expression in healthy tissues. To ensure an ADC’s efficacy, two important things have to be considered. One element is the linker between the antibody and the payload as it should ensure that the payload is not released prematurely to avoid systemic toxicity. The second element is a powerful cytotoxic agent as payload linked to the antibody characterized by a high therapeutic index. The design of the linker determines the drug release from the antibody. Cleavable and non-cleavable linkers are used depending on the different payloads. ADCs should reduce off-target toxicities by limiting the exposure of the payload to normal tissues. In addition to the direct therapeutic effect of ADCs, which is amongst others dependent on distribution and antigen expression, some ADC payloads also work by the bystander killing effect, e.g. trastuzumab deruxtecan (T-DXd) as described below, where the payload diffuses from targeted to untargeted cells, depending on the chemistry of the drug [2–5].

BC is a heterogenous group of malignancies with a broad range of histopathological characteristics. Fortunately, most BC diagnoses are made at an early stage in which curative treatment is still the primary goal. In the metastatic setting, treatment becomes increasingly difficult with the emergence of primary or secondary drug resistance, and this is particularly true for advanced triple-negative BC (TNBC). Currently, three ADCs received approval by the US Food and Drug Administration (FDA) and are in routine clinical use; many more ADCs are in earlier and later stages of development, and their future approval will improve treatment options for patients with advanced but potentially also early-stage BC over time. Trastuzumab emtansine (T-DM1), was the first ADC getting approval for early and advanced human epidermal growth factor receptor 2 (HER2)-positive BC. T-DM1 is currently approved for the use in patients with unresectable or metastatic HER2-positive BC who were already pretreated with trastuzumab and taxane or relapsed during or within 6 months after completement of adjuvant therapy based on the results of the EMILIA trial [6]. For early HER2-positive BC, T-DM1 is approved as adjuvant treatment in patients having residual invasive disease in the breast and/or lymph nodes after neoadjuvant treatment with at least a taxane and trastuzumab based treatment regimen as investigated in the KATHERINE trial [7]. Recently, T-Dxd was approved for patients with HER2-positive unresectable or metastatic BC, who received at least two prior anti-HER2-directed treatments, based on the results of the DESTINY-Breast01 trial [8] (Table 1). For TNBC, sacituzumab govitecan (SG) is now approved for patients with unresectable or metastatic TNBC who received two or more prior therapies with at least one of them for advanced disease as investigated in the ASCENT trial [9] (Table 1). In this review, we are focusing on the newer ADCs including T-DXd and SG, their pharmacology, mechanism of action, their early-stage trials and registration studies. Finally, we would like to give an overview on the latest results of some newer ADCs in further stages of development.

**Table 1.** Results of the completed phase 3 studies for T-DXd [16], trastuzumab duocarmazine [28] and SG [9]

**Study**		**Comparators**	**Study population**	**Primary outcome**	**Important results**
T-DXd	DESTINY-Breast03 (*n* = 524 )	T-DXd *versus* T-DM1	HER2-positive, unresectable and/or MBC, previously treated with trastuzumab and taxane	PFS (BICR)	HR 0.2840 (PFS not reached and 6.8 months, *P* = 7.8 × 10^−22^)
Trastuzumab duocarmazine	TULIP (*n* = 437 )	Trastuzumab duocarmazine *versus* treatment of physician’s choice	HER2-positive, locally advanced or MBC, ≥ 2 previous MBC regimens or previous treatment with T-DM1	PFS (BICR)	HR 0.64 (PFS 7.0 and 4.9 months, *P* = 0.002 )
SG	ASCENT (*n* = 468 )	SG *versus* single-agent chemotherapy of physician’s choice	TNBC relapsed or refractory to ≥ 2 previous standard chemotherapy for advanced or MBC, taxane must be included in the previous therapies	PFS (BICR)	HR 0.41 (5.6 and 1.7 months, *P* < 0.001 )

MBC: metastatic breast cancer; PFS: progression-free survival ; BICR: blinded independent central review; HR: hazard ratio

## T-DXd

### Pharmacology and mechanism of action

T-DXd (also known as DS-8201a) is a recently FDA-approved ADC based on a trastuzumab like anti-HER2 antibody, a cleavable tetrapeptide linker and a novel topomerase I inhibitor, exatecan derivative (DX-8951 derivative, DXd). Ogitani et al. [10] evaluated the *in vitro* and *in vivo* pharmacologic activity of T-DXd in comparison to T-DM1 in several HER2-positive cell lines and patient-derived xenograft (PDX) models [10]. Binding activity and antibody-dependent cellular cytotoxic activity of T-DXd is similar with the unconjugated anti-HER2 antibody. T-DXd inhibits Akt phosphorylation and induces phosphorylation of Chk1 and Histone H2AX as markers of DNA damage. In cynomolgus monkeys, the highest non-severely toxic dose of T-DXd was shown to be 30 mg/kg, suggesting high relative tolerability in humans. Of interest, Ogitani et al. [11] could also show effectiveness of T-DXd in T-DM1-insensitive PDX-models with high as well as with low HER2-expression [11]. A mechanism of action in addition to T-DXds direct cytotoxic effect might be a so called bystander killing effect. In comparison to T-DM1, T-DXd’s cytotoxic payload is highly membrane-permeable. In a coculture condition of HER2-positive and HER2-negative cells, T-DXd kills both HER2-positive and HER2- negative BC cells, whereas T-DM1 did not. This was further investigated using an *in vivo* xenograft mice model [female CAnN.Cg-Foxn1 ^nu^ /CrlCrlj mice (BALB/c nude mice)] and it could be shown that T-DXd’s bystander killing effect is limited to HER2-negative cells directly neighboring HER2-positive cells, so that a systemic toxicity due to this effect seems to be of low concern.

### Early stage clinical trials

T-DXd was investigated in different trials, not only limited to BC patients, but also focusing on non-breast HER2-positive solid tumours such as gastric cancer. In a phase 1 dose-escalation study, patients with advanced BC were included together with gastric or gastro-oesophageal cancer patients [12]. Twenty-four patients with breast, gastric or gastro-oesophageal carcinomas were included at two study sites in Japan regardless of their HER2-status. In this study, the maximum tolerated dose of T-DXd was not reached, a dose of 5.4 or 6.4 mg/kg body weight was recommended based on safety and activity. In another phase 1 trial, patients with HER2-positive advanced BC who had previously been treated with T-DM1 were investigated in 14 centers (eight in the USA and six in Japan) [13]. A total of 115 patients received at least one dose of T-DXd, with the primary endpoint of the trial being safety and preliminary signals of activity. Interestingly, all patients had at least one treatment-related adverse event. Besides hematotoxicity, adverse effects affecting the lung were reported in 20 cases including interstitial lung disease (ILD), pneumonitis and organizing pneumonia, and 2 patients in the study died due to pneumonitis. More than half of the patients in this heavily pretreated group experienced a confirmed radiological response. Another phase 1b trial investigated whether T-DXd is also active in HER2-low advanced or metastatic BC [immunhistochemistry (IHC) 1+ or 2+/*in situ* hybridization–] when given at the recommended dose [14]. Fifty-four mostly heavily pretreated (median 7.5 prior therapies) patients were enrolled. Twenty out of 54 patients (37%) showed a confirmed radiological response [95% confidence interval (CI) 24.3 to 51.3 months] by independent central review with a median duration of response of 10.4 months (95% CI 8.8 months to not evaluable). Treatment-emergent adverse events of grade 3 or higher were seen in 34/54 patientes (63.0%) including neutrophil count decrease, white blood cell count decrease, anemia, hypokalemia, platelet count decrease, aspartate aminotransferase (AST) increase, decreased appetite, febrile neutropenia, cellulitis and diarrhea (each occurring in ≥ 5% of patients) with 3 fatal events due to ILD. In conclusion, early clinical trials of T-DXd showed very promising antitumor activity in BC patients with both HER2-positive and as well some with HER2-negative disease with a substantial proportion of patients suffer moderate to severe toxicity, particularly cases of T-DXd-associated, potentially fatal ILD.

### Registration studies and more recent data

The approval of T-DXd by the FDA was based on the DESTINY-Breast01 trial published by Modi et al. [8] in the year 2020. In this phase 2 study, T-DXd was evaluated in T-DM1 pretreated patients with HER2-positive metastatic BC. In the first part of the trial, 3 different doses of T-DXd were tested to evaluate the recommended dose. In the second part, 184 patients with HER2-positive BC received the recommended dose of T-DXd at 5.4 mg/kg. At the recommended dose of 5.4 mg/kg, 112 out of 184 patients (60.9%) exhibited a confirmed radiological response (95% CI 53.4 to 68), with a median response duration of 14.8 months (95% CI 13.8 to 16.9) in this extensively pretreated patients (median of six prior systemic treatments for metastatic disease). PFS was 16.4 months (95% CI 12.7 to not reached). Severe frequently reported side effects of grade 3 or higher were neutropenia in 38 patients (20.7%), anemia in 16 patients (8.7%) and nausea in 14 patients (7.6%). T-DXd-associated ILD remained an issue, with 25 patients (13.6%) experiencing any-grade ILD. In December 2020, Modi et al. [15] presented an update on the trial showing a generally tolerable safety profile consistent with previous presented results [15]. In the longer follow-up period, 5 deaths attributed to ILD (2.7%) were reported. The results of this study led to the accelerated approval of T-DXd by the FDA in December 2019 for advanced, unresectable or metastatic HER2-positive BC pretreated after at least 2 prior HER2-targeted therapies in the metastatic setting. In the meantime, T-DXd has also been approved by the European Medicines Agency (EMA) and by Swissmedic.

Recently, results of the DESTINY-Breast03 were presented at the presidential session of the 2021 ESMO Annual Meeting [16] (Table 1). In this phase 3 study, T-DXd was randomized against T-DM1 in patients with HER2-positive, metastatic BC previously receiving trastuzumab and a taxane. In total, 524 patients with HER2-positive advanced BC were randomized, and the primary endpoint of PFS was significantly improved with T-DXd compared to T-DM1 [PFS by BICR, HR 0.2840, 95% CI 0.2165 to 0.3727, *P* = 7.8 × 10^−22^ (two-sided)], median PFS was not reached for T-DXd and was 6.8 months for T-DM1. The 12-months PFS rate was 75.8 (95% CI 69.8 to 80.7) for T-DXd and 34.1% (95% CI 27.7 to 40.5) for T-DM1. The 12-months overall survival (OS) was 94.1% with T-DXd and 85.9% for T-DM1 [HR 0.5546, 95% CI 0.3587 to 0.8576, *P* = 0.007172 (did not cross pre-specified boundary for significance)]. Median treatment duration also favoured T-DXd with 14.3 months *versus* 6.9 months for T-DM1. Overall treatment-associated toxicity was similar in both treatment arms. Treatment-associated ILD occurred in 10.5% of patients of the T-DXd arm, but no grade 4 or 5 ILD was reported, as investigators supposedly improved the awareness and management of T-DXd-associated pulmonary toxicity by introducing proactive radiological assessments.

## SG

### Pharmacology and mechanism of action

SG is a new ADC consisting of a trophoblast cell-surface antigen 2 (Trop-2) antibody linked to a highly potent, active metabolite of irinotecan (SN-38). TNBC is often associated with a poor prognosis and the therapeutic options have been limited due to missing targets such as HER2 or hormone receptors. Trop-2 however can be found in the majority of epithelial carcinomas including BC [17]. Trop-2 is also expressed in many healthy tissues of the body, but with expression levels substantially below what is typically found in malignant epithelial tumors, making Trop-2 an excellent target for anticancer drug treatment [18]. SN-38, the active metabolite of irinotecan, inhibits topoisomerase I similar to the exatecan derivative in T-DXd, and results in cell death due to DNA-breakage by inhibiting the re-ligation of the DNA-strand [19]. SN-38 is a promising toxic payload for ADCs as the treatment with irinotecan as a pro-drug is associated with some pharmacokinetic issues such as a low conversion into the active drug SN-38 and rapid clearance from the blood [20]. In a TNBC-xenograft model using NCr female athymic nude (nu/nu) mice, SG showed a significantly increased tumor regression in comparison to mice treated with irinotecan [17]. After treatment with SG, intratumoral concentrations of SN-38 were 20- to 136-fold higher than after infusion of irinotecan in a mouse [NCr female athymic nude (nu/nu) mice] xenograft study [21].

### Early stage clinical trials

SG was first investigated by Bardia et al. [22] in a phase 1/2 basket-trial including patients with advanced epithelial cancers [22]. In this clinical study, 108 patients with a minimum of 2 prior therapies for metastatic TNBC received SG at 10 mg/kg on days 1 and 8 of a 3-week cycle. Neutropenia/decreased neutrophil count was the most common adverse event of grade 3 or 4 occuring in 45 patients (42%). Febrile neutropenia of grade 3 and 4 was observed in 9 patients (8%). In eligible TNBC, 36 patients experienced a radiological response, including 33 patients (34.3% by BICR, 95% CI 25.4 to 44) with a partial response and 3 patients with a complete response. Median duration of response was 9.1 months (95% CI 4.6 to 11.3). Median PFS was 5.5 months (95% CI 4.1 to 6.3) and OS was 13.0 months (95% 11.2 to 13.7). In conclusion, SG showed durable responses in patients with heavily pretreated, metastatic TNBC. Furthermore, a prespecified hormone receptor-positive and HER2-negative subgroup from the above mentioned basket trial was analysed [23]. Among 54 women with estrogen receptor (ER)-positive advanced BC progressing after endocrine treatment and chemotherapy, 17 women (31.5%) experienced a radiological response (95% CI 19.5 to 45.6, 17 partial responses), with a median duration of response of 8.7 months (95% CI 3.7 to 12.7) and a median PFS of 5.5 months (95% CI 3.6 to 7.6) and a median OS of 12 months (95% CI 9.0 to 18.2).

### Registration studies

The results of the phase 3 ASCENT trial by Bardia et al. [9] led to the regular approval of SG by the FDA [9] (Table 1). SG has been recently approved by the EMA and by Swissmedic as well. In this randomized phase 3 trial, SG was randomized against physician’s choice using single-agent chemotherapy in 468 patients with relapsed or refractory TNBC without brain metastases. Median PFS was 5.6 months in the SG group and 1.7 months in the control group (HR 0.41, 95% CI 0.32 to 0.52, *P* < 0.001). Median OS also favoured SG with 12.1 months and 6.7 months in the control group (HR 0.48 for death, 95% CI 0.38 to 0.59, *P* < 0.001). A radiological response was experienced by 35% of patients in SG group *versus* 5% in the chemotherapy group. Adverse events of grade 3 or higher were more common in SG-treated patients. The most reported adverse event was neutropenia in 132 patients in the SG-treated patients (51%) and in 74 patients in the chemotherapy group (33%). Leukopenia [26 patients (10%) and 12 patients (5%)], diarrhea [27 patients (10%) and 1 patient (< 1%)], anemia [20 patients (8%) and 11 patients (5%)] as well as febrile neutropenia [15 patients (6%) and 5 patients (2%)] were reported frequently as well. In a biomarker analysis of the ASCENT trial, patients with high or medium Trop-2 expression benefit from SG in comparison to standard-of-care chemotherapy regardless of their *BRCA1/2* mutation status [24]. Because of the small number patients in the low Trop-2 expression subgroup definitve conclusions cannot be made. Another subgroup analysis reported by Diéras et al. [25] could show that in patients with brain metastases in the ASCENT trial (*n* = 61) SG was numerically better than treatment of physician’s choice regarding tumor response and PFS but not OS, but data interpretation has to be done careful due to the small sample size [25]. In conclusion, the ASCENT trial established SG as a later-line systemic treatment in patients with advanced, pretreated TNBC.

## New ADCs in later stages of development to have on the list

### Trastuzumab duocarmazine

Trastuzumab duocarmazine (also known as SYD985) is another HER2-targeted ADC consisting of an antibody based on trastuzumab that is covalently bound to a linker-drug based on duocarmycin [26]. In a phase 1 dose escalation and expansion study by Banerji et al. [27], 39 patients received trastuzumab duocarmazine at escalating doses, and the recommended phase 2 dose was set at 1.2 mg/kg [27]. In the dose-expansion phase of this trial, 146 patients were treated at the recommended phase 2 dose. Treatment-related serious adverse events were reported in 16/146 patients (11%), and the most common treatment-related adverse events of any grade included fatigue (33%), conjunctivitis (31%) and dry eyes (31%). Ocular adverse events occurred in 104 out of the 146 (71%) patients, and 10 events (7%) were grade 3. No deaths were caused by trastuzumab duocarmazine. An objective response according to the response evaluation criteria in solid tumors (RECIST) was achieved in 16 out of 48 (33%) patients in the BC dose-expansion cohorts. Nine out of 32 (28%) with HER2-low, hormone receptor-positive BC as well as 6 out of 15 patients (40%) with HER2-low, hormone receptor-negative BC achieved an objective response. Recently, the primary outcome of the phase 3 TULIP trial was reported at the 2021 ESMO Annual Meeting by Saura Manich et al. [28] (Table 1). The efficacy of trastuzumab duocarmazine in locally advanced or metastatic HER2-positive BC in patients with ≥ 2 prior metastatic BC treatments or previous treatment with T-DM1 in a 2:1 randomization compared to physician’s treatment of choice was evaluated in 437 patients (median age 56 years, median number of 4 prior treatments). Median PFS (central review) was 7.0 months *versus* 4.9 months in favor of the trastuzumab duocarmazine group (HR 0.64, CI 0.49–0.84, *P* = 0.002). The first OS analysis showed an HR of 0.83 (CI 0.62–1.09, *P* = 0.153). Ocular events including conjunctivitis (38.2%) and keratitis (38.2%) as well as fatigue (33.3%) were the most frequently reported adverse events. Adverse events leading to discontinuation of treatment in the trastuzumab duocarmazine group (35.4%) were eye disorders (20.8%) and respiratory disorders (6.3%). Due to the significantly improved PFS, trastuzumab duocarmazine may enter the BC field as a new treatment option for patients with heavily pretreated locally advanced or metastatic HER2-positive BC in the future.

### Disitamab vedotin

Another ADC to have on the list is disitamab vedotin (also known as RC48-ADC). This ADC is consisting of disitimab, an HER2-targeting humanized mAb, conjugated with monomethyl auristatin E (MMAE), a microtuble-disrupting agent, by a protease cleavable linker. Superior anti-tumor activity compared to T-DM1 was shown for this new ADC in xenograft models [29]. In a phase 1 and phase 1b study reported by Xu et al. [30], good tolerability and promising efficacy was shown for disitimab vedotin in locally advanced or metastic HER2-positive BC patients [30]. The benefit risk ratio favoured a dose of disitamab vedotin of 2.0 mg/kg administered every two weeks in patients with advanced BC. In the study by Xu et al. [30], 22 out of 70 patients (31.4%) showed a radiological response. The median PFS was 5.8 months. The most common reported treatment-related adverse events (mostly classified as grade 1 or 2) included increased AST (62.9%) and alanine aminotransferase (ALT) (61.4%), leukopenia (51.4%), hypoesthesia (51.4%) and neutropenia (51.4%). Grade 3 or 4 events were reported in 41.4% of the patients (29 patients) and include neutropenia (21.4%), asthenia (15.7%) and leukopenia (10.0%). Currently, a phase 2/3 study is evaluating the efficacy of disitimab vedotin *versus* capecitabine and lapatinib in HER-positive locally adcvanced metastatic BC. Furthermore, the activity of disitamab vedotin is evaluated in HER2-positive advanced BC with liver metastases (ClinicalTrials.gov identifier: NCT03500380). These studies are still open for enrollment. A phase 3 study is currently evaluating the efficacy and safety of disitimab vedotin for the treatment of locally advanced or metastatic BC with low HER2-expression (ClinicalTrials.gov identifier: NCT04400695).

### Ladiratumab vedotin

Ladiratumab vedotin (also known as SGN-LIV1A) is an ADC consisting of a humanized antibody targeting the zinc transporter LIV-1 and is linked through a proteolytically cleavable linker to MMAE. LIV-1 is expressed in both ER-positive BC as well as TNBC, but can also be found in other entities such as prostate, ovarian and uterine cancer as well as in melanoma [31]. In an ongoing phase 1 study by Modi et al. [32] ladiratumab vedotin administered every 3 weeks is evaluated in women with LIV-1-positive, unresectable, locally advanced or metastatic BC who had at least ≥ 2 cytotoxic treatment for advanced or metastatic disease [32]. At the time of last study report, 69 patients (18 hormone receptor-positive/HER2-negative, 51 triple-negative) received a median of 3 cycles of ladiratumab vedotin every 3 weeks, and no dose-limiting toxicities occured in the 19 evaluable patients. The expansion cohorts were opened to TNBC patients at 2.0 and 2.5 mg/kg every 3 weeks. Most frequent treatment-emergent adverse events included fatigue (59%), nausea (51%), peripheral neuropathy (44%) and alopecia (36%). Decreased appetite (33%), constipation (30%), abdominal pain (25%), diarrhea (25%) and neutropenia (25%) occured often as well. Most of the events were grade 1 or 2. There was one treatment-related death due to neutropenic fever with sepsis in a patient receiving a dose exceeding 200 mg per cycle. The disease control rate was 59% (10 stable diseases) in the 17 efficacy evaluable hormone receptor-positive/HER2-negative patients in the dose escalation phase. The disease control rate was 64% in the 44 TNBC patients from the dose escalation and expansion cohort (14 partial responses, 14 stable disease) concomitant with an objective response rate of 32%, confirmed partial response rate of 21% and a clinical benefit rate of 36%. In the same report, median PFS was 11.3 weeks in the TNBC cohort (95% CI 6.1 to 17.1). Enrollment for the triple negative dose expansion cohort is still ongoing. Just recently at the ESMO congress 2021, results were presented for the efficacy and safety of every 1 week administered ladiratumab vedotin in 81 patients in first and second line endocrine therapy refractory hormone receptor-positive/HER2-negative as well as second line metastatic TNBC [33]. Subsequent enrollment was only continued for the 1.25 mg/kg every 1 week cohort, although no dose limiting toxicities were reported; 6 patients (66%) in the 1.5 mg/kg-cohort were reported with neutropenia (3 of them with grade 4 neutropenia). The confirmed objective response rate was 28% (95% CI 13 to 47) in the 29 patients with second line TNBC receiving 1.25 mg/kg. Further investigation is underway. In the I-SPY 2 trial, neoadjuvant ladiratumab vedotin was tested in women with high-risk stage II/III BC with pathological complete remission (pCR) as primary endpoint in comparison with paclitaxel weekly for 12 weeks and four cycles ofdoxorubicin/cyclophosphamid chemotherapy in both study arms. Ladiratumab vedotin was comparable to paclitaxel with regard to pCR with similar side effects, at least with less peripheral neuropathy [34]. As both ADCs as well as checkpoint inhibitors are emerging in the treatment of TNBC, the combination of pembrolizumab and ladiratumab vedotin is evaluated in a more recent ongoing phase 1b/2 study. Patients with TNBC were included and were not pre-selected for their programmed death-ligand 1 (PD-L1) or LIV-1 expression. Patients were treated first-line for TNBC. Fifty-one patients were evaluable for safety and 44 patients (86%) reported treatment-emergent adverse events, most frequently nausea (53%), fatigue (45%), diarrhea (43%) and alopecia (33%). Constipation, hypokalemia, vomiting, decreased appetite, abdominal pain, decreased weight, neutropenia and peripheral neuropathy occured in 20–30% of patients. In the 26 patients followed for at least 3 months, the confirmed objective response rate was 54% (95% CI 33.4 to 73.4) [35].

## Conclusions

ADCs such as SG and T-DXd as well as the other ADC in later stages of development with different targets will change the treatment landscape of BC as well as of other cancer entities. Through targeting a special antigen, the payload, which otherwise would be too toxic in a systemic therapy, can bring a maximum of toxicity to the cancer cells with a minimum of adverse effects. Some of the additional effects, such as the bystander killing effect, are useful and part of their mechanism of action and could potentially broaden the target population of the drug. Therefore drugs, that otherwise could not be administered, can be administered now and offer new treatment options to heavily pretreated patients. The efficacy is promising with manageable but very different, side effects. In patients getting T-DXd, especially the lung function should be monitored thoroughly as ILD or other pulmonal side effects might be fatal. The results from the DESTINY-Breast03 trial revealed that T-DXd improved PFS by 72% over T-DM1 and doubled response rates [16]. Similarly, the results of the TULIP trial, comparing trastuzumab duocarmazine *versus* physician’s treatment of choice in pretreated HER2-positive metastatic BC, suggest that this ADC may be a novel therapeutic modality for such patients [28]. Likewise, the ASCENT trial, exploring SG *versus* single-agent chemotherapy of physician’s choice in previously treated women with metastatic BC showed a survival benefit [9]. Hopefully, these findings will encourage participation in further randomized clinical trials, especially among patients with metastatic BC or at high risk for refractory BC.

Ongoing trials are currently investigating SG, T-DXd as well as the other ADCs in earlier treatment lines and different therapy settings in BC as well as in other cancer entities, as single agent or in combination, and the results are anticipated to change the treatment not only of BC but of other cancer entities as well. Future developments include the investigation of combinations with checkpoint inhibitors, tyrosine kinase inhibitors as well as the combination of anti-HER2-directed treatments. With regard to checkpoint inhibitors, T-DXd is currently, amongst others, tested in combination with durvalumab (ClinicalTrials.gov identifier: NCT04538742, NCT04556773), nivolumab (ClinicalTrials.gov identifier: NCT03523572) and pembrolizumab (ClinicalTrials.gov identifier: NCT04042701) in different treatment settings. SG is, amongst others, investigated in combination with pembrolizumab (ClinicalTrials.gov identifier: NCT04468061, NCT04448886, NCT04230109), atezolizumab (ClinicalTrials.gov identifier: NCT04434040, NCT03424005) and avelumab (ClinicalTrials.gov identifier: NCT03971409). In conclusion, ADCs are a promising new therapeutic approach in heavily pretreated patients in BC as well as in other cancer entities and further study results are eagerly awaited.

## Abbreviations

ADCs: antibody-drug conjugates
BC: breast cancer
BICR: blinded independent central review
CI: confidence interval
FDA: Food and Drug Administration
HER2: human epidermal growth factor receptor 2
HR: hazard ratio
ILD: interstitial lung disease
MBC: metastatic breast cancer
OS: overall survival
PFS: progression-free survival
SG: sacituzumab govitecan
T-DM1: trastuzumab emtansine
T-DXd: trastuzumab deruxtecan
TNBC: triple-negative breast cancer
Trop-2: trophoblast cell-surface antigen 2

## Declarations

### Copyright

© The Author(s) 2022.
